# Associations between urinary glycosaminoglycans and onset of acute respiratory distress syndrome in sepsis patients: a prospective exploratory study

**DOI:** 10.1038/s41598-025-02109-5

**Published:** 2025-05-22

**Authors:** Ziyuan Shen, Zhengying Li, Hua Zhang, Xiaoyun Wei, Senhao Wei, Feng Zhao, Cui Yang, Zhukai Cong, Zhongnan Yin, Chenchen Ding, Cai Tie, Xi Zhu

**Affiliations:** 1 Department of Critical Care Medicine, Peking University Third Hospital, Beijing, 100191 China; 2 School of Chemical and Environmental Engineering, China University of Mining and Technology-Beijing, Beijing, 100083 China; 3 State key laboratory Coal resources and Safe Mining, China University of Mining and Technology-Beijing, Beijing, 100083 China; 4 Center of Epidemiology, Peking University Third Hospital, Beijing, 100191 China; 5 Biobank, Peking University Third Hospital, Beijing, 100191 China

**Keywords:** Glycosaminoglycans, Inflammatory factors, Sepsis, Acute respiratory distress syndrome, Risk factors, Predictive markers, Respiratory distress syndrome

## Abstract

The aim of the current study was to explore the association between urinary glycosaminoglycans and the onset of acute respiratory distress syndrome (ARDS) in sepsis patients. We conducted a prospective cohort study of 49 sepsis patients and collected clinical characteristics, urine, and blood samples within 24 h of admission to the Surgical Intensive Care Unit (SICU) of Peking University Third Hospital. Samples from patients who did not develop ARDS were collected from day 1 to day 7 after SICU admission, while samples from patients who developed ARDS were collected from day 1 to the day of onset, forming time-series data. A liquid chromatography-mass spectrometry (LC-MS) system was used to determine urinary dermatan sulfate (DS), chondroitin sulfate (CS), and heparan sulfate (HS) concentrations; enzyme-linked immunosorbent assay (ELISA) and cytometric bead array (CBA) were used to measure plasma inflammatory factors. The endpoint was the onset of ARDS. We used generalized estimating equations to identify associations between urinary glycosaminoglycans and ARDS onset in sepsis patients. Twenty-two of the 49 sepsis patients developed ARDS, and 132 plasma samples and 132 urine samples were obtained. Between the ARDS and non-ARDS groups, sex, pneumonia, Acute Physiology and Chronic Health Evaluation II (APACHE II), Lung Injury Prediction Score (LIPS), and PaO_2_/FiO_2_ showed statistically significant differences. Generalized estimating equations showed that DS and tumor necrosis factor-α (TNF-α) were independently associated with ARDS onset in sepsis patients, but their generalizability across ARDS subtypes warrants further validation.

**Supplementary Information:**

The online version contains supplementary material available at 10.1038/s41598-025-02109-5.

## Introduction

Sepsis is defined as the host’s uncontrolled response to infection leading to organ dysfunction that endangers the patient’s life^1^, which is a common cause of acute respiratory distress syndrome (ARDS). The heterogeneity of ARDS, lack of specific diagnostic criteria and therapies, and rapid progression after diagnosis lead to high mortality ranging from 35–46%^2^. Currently, cytokine storm is considered an important pathophysiological mechanism for ARDS development, and thus increasing numbers of studies are attempting to use clinical parameters and blood inflammatory factors to predict or diagnose ARDS. In addition, there is an urgent need to discover new biomarkers that are more representative and predictive in value.

Disruption of the glycocalyx in the development of ARDS is also receiving increasing attention. The glycocalyx is a gel-like layer covering the luminal surface of endothelial cells^3^, which is composed of proteoglycans, glycosaminoglycans (GAGs), and glycoproteins. It also adheres circulating proteins like albumin to form the endothelial surface^4^. A large number of basic and clinical studies have demonstrated that the glycocalyx plays important roles in maintaining vascular permeability, sensing fluid shear forces^5^, preventing microthrombosis^6^ and regulating leukocyte adhesion^7^. The glycocalyx is modulated by endothelial stabilizing agents, physical forces on the vascular wall, and inflammatory factors^8^. In addition, the glycocalyx is also found to cover epithelium. Researchers have found that alveolar epithelial glycocalyx degradation disrupts surfactant function^9^. Changes in vascular permeability caused by degradation of the glycocalyx in alveolar capillary endothelium lead to bilateral pulmonary edema, ultimately resulting in ARDS.

**Fig. 1:**
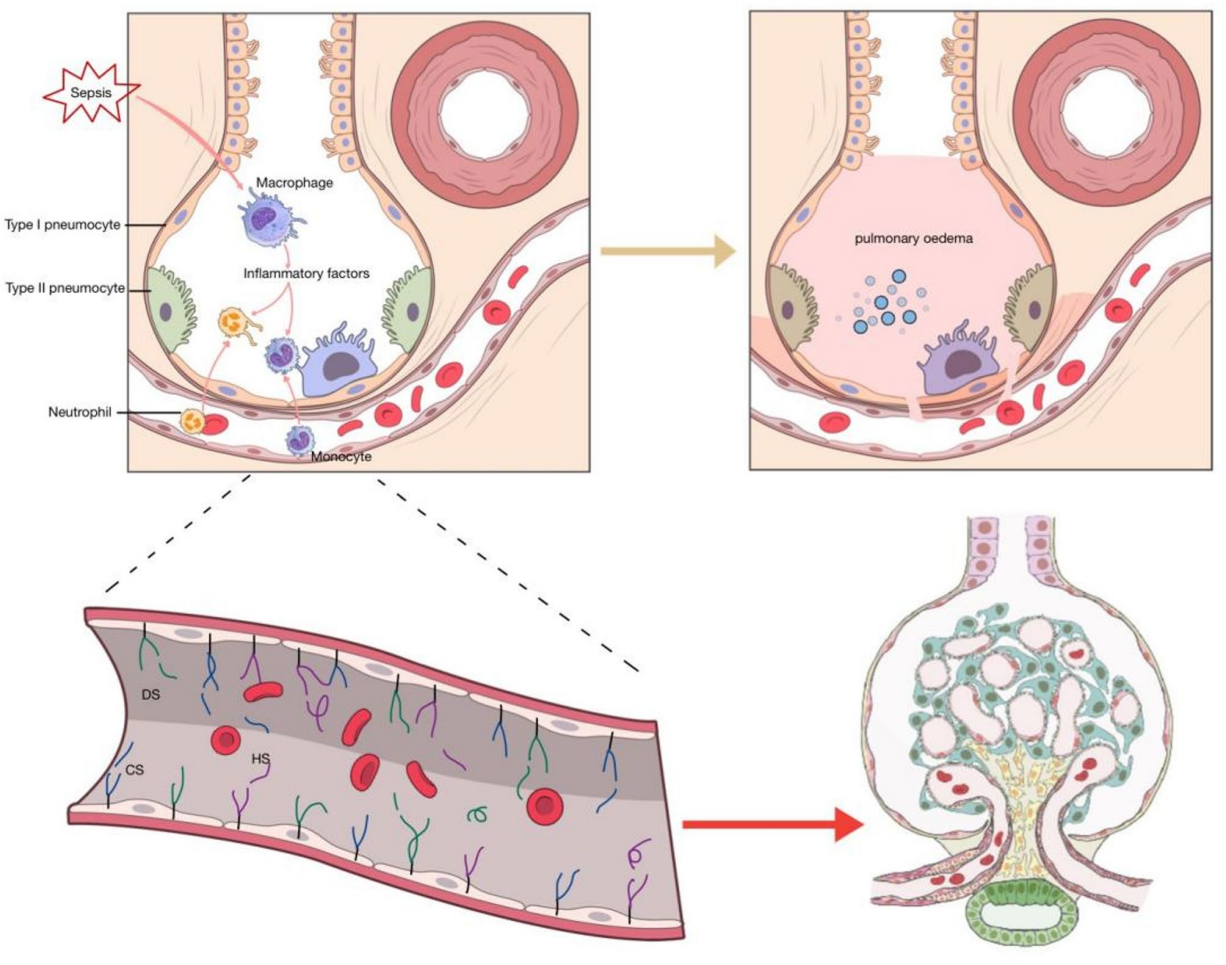
Endothelial GAG shedding disrupts the alveolar-capillary barrier, causing pulmonary edema, with shed GAGs excreted in urine.This map was generated by NIAID Visual & Medical Arts. (2024/10/8). Alveoli. NIAID NIH BIOART Source. bioart.niaid.nih.gov/bioart/14; NIAID Visual & Medical Arts. (2024/10/8). Blood Vessel. NIAID NIH BIOART Source. bioart.niaid.nih.gov/bioart/55; Human Reference Atlas. (2024/12/19). Renal Corpuscle. NIAID NIH BIOART Source. bioart.niaid.nih.gov/bioart/562.

GAGs are important components of the glycocalyx, which consist of repeating disaccharide units and are covalently attached to proteins to form proteoglycans. According to the different disaccharide units, GAGs are divided into chondroitin/dermatan sulfate (CS/DS), heparin/heparan sulfate (Hep/HS), keratan sulfate (KS), and non-sulfated hyaluronan (HA). Except for KS, which consists of hexosamine and galactose, others are all composed of hexosamine and hexuronic acid. Besides, the number and position of the sulfate residues, and the presence of N-acetyl and/or N-sulfate groups contribute to the diversity of GAGs^10^. According to Rizzo’s research, in ARDS, airspace fluid GAGs concentrations correlate with lung injury severity and disease course^9^. Queisser found that in COVID-19, fragments of GAGs were released into the blood circulation, which could further aggravate the disease^11^. Carmichael showed that elevations in circulating GAGs were positively correlated with postoperative Sequential Organ Failure Assessment (SOFA) scores at 24 h in intra-abdominal sepsis patients^12^. Circulating GAGs are filtered by the nephrons and excreted in the urine (showed in Fig. 1). Schmidt confirmed that urinary GAGs had predictive value for disease progression in sepsis and ARDS patients^13^. However, no studies have yet demonstrated whether there is a correlation between urinary GAGs and the occurrence of ARDS. Therefore, in this study, we aim to investigate whether urinary GAGs are associated with the subsequent occurrence of ARDS by measuring urinary GAGs levels in septic patients and tracking whether they develop ARDS and the time of onset, which may lay a foundation for identifying novel biomarkers for predicting ARDS.

## Methods

The study was reviewed and approved by the Institutional Review Board of Peking University Third Hospital (Beijing, China) (ethics number M2020278) and was performed in accordance with relevant guidelines and regulations. Written informed consent was obtained from each patient or their relative.

### Study population

The study was performed in a 20-bed surgical intensive care unit (SICU) of Peking University Third Hospital (Beijing, China) between January 2022 and December 2022. The study design, performance, and report complied with the Standards for Reporting of Diagnostic Accuracy guidelines^14^. Sepsis patients enrolled in our study comprised two categories: (1) patients who developed secondary sepsis following surgery (e.g., intra-abdominal infections after gastrointestinal surgery); (2) patients with primary sepsis who have undergone surgical intervention (e.g., suppurative cholangitis, intestinal perforation).They were monitored and treated following international guidelines for management of sepsis and septic shock^15^.

The patients with sepsis who stayed in the SICU longer than 48 h were prospectively and consecutively enrolled. The exclusion criteria included: (1) patients without consent; (2) age < 18 years; (3) chronic kidney disease; (4) organ or bone marrow transplantation; (5) receiving mannitol or heparin treatment; (6) developed ARDS before admission; and (7) more than 30% missing data.

### Sample collection

**Fig. 2:**
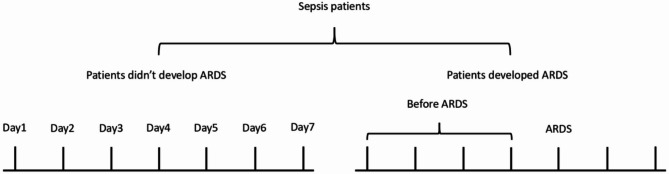
Days of sample collection in sepsis patients. Patients didn’t develop ARDS: Day1-Day7; Patients developed ARDS: Day1-The day of onset.

Blood and urine were collected within 24 h of admission to the SICU. Samples from patients who did not develop ARDS were collected from day 1 to day 7 after SICU admission, while samples from patients with ARDS were collected from day 1 to the day of onset. Days of sample collection are shown in Fig. 2.

### Materials

MS-grade water, acetonitrile, Acetic acid, methanol (Fisher Scientific, USA).DS, CS and HS standards (Sigma-Aldrich, USA), CBA kits (Biolegend, China), ELISA kits (Abebio, China).

### Bio-markers measurements

#### Urinary GAGs testing: pretreatment of bio-samples

500 µl of urine samples were centrifuged at 15,000/g for 5 min,15 µl supernatant was transferred to a Polymerase Chain Reaction(PCR) tubes and dried with vacuum concentrator. 100 µl of 1.25 mol/l methanol hydrochloride was added into each tube, incubation was carried out at 65 °C for 5 h.The reaction solvent was concentrate dried with vacuum concentrator. The dried samples were stored at – 20 °C, prior to Ultra High-Performance Liquid Chromatography - Multiple Reaction Monitoring (UHPLC-MRM) analysis. The dried samples were reconditioned with 100 µl acetonitrile/water (90/10,v/v) and injected directly.

#### Urinary GaGs testing: UHPLC-MRM analysis

DS, CS and HS analysis were performed on the LC-MS system consisting of a SCIEX Triple Quad 5500 + MS with an ESI source (MA, USA) and a Thermo Scientific Dionex Ultimate 3000 HPLC (MA, USA). A Waters ACQUITY UPLC BEH amide (2.1 × 75 mm) was adapted. Mobile phase A was 10 mM acetic acid aqueous solution, and mobile phase B was acetonitrile. Elution conditions are shown in Supplementary Table 1. The flow rate was 0.3 ml/min. The column temperature was set as 30 °C. The injection volume was 5 µl. Data acquisition was performed with positive-mode.

### Plasma inflammatory factors testing

Acquired blood samples were rested for 30 min and subsequently centrifuged at 2500 rpm at 4 °C for 10 min, and supernatant plasma was stored and frozen at – 80 °C until used. Plasma concentrations of Interferon-γ (IFN-γ),Tumor necrosis factor-α (TNF-α), Monocyte chemoattractant protein-1 (MCP-1), Interleukin-6 (IL-6), Interleukin-8 (IL-8),Interleukin-10 (IL-10) were measured by commercial CBA kits (Biolegend, China), and we used ELISA kits (Abebio, China) to measure Angiopoietin II (Ang II), Krebs von den Lungen-6 (KL-6), Receptor for advanced glycation end products (RAGE), following the manufacturer’s protocol.

The biomarkers were measured by technicians of the Biobank of Peking University Third Hospital who were blinded to clinical data, and the physicians in charge were blinded to the biomarker test results.

### Clinical endpoints and definition

All patients with sepsis were diagnosed by Sepsis-3 criteria in 2016^1^, and ARDS was diagnosed according to the Berlin Definition in 2012^16^. The primary endpoint was the onset of ARDS within 7 days after enrollment.

The primary endpoint was determined by two experienced clinicians who were blinded to the expression of the urinary GAGs or plasma inflammatory factors. If there was any objection, a third clinician was invited to assist in the diagnosis. If still not certain, the diagnosis was made again 4–6 h later until the diagnosis was clear or excluded.

### Clinical data extraction

All clinical data were prospectively collected on the basis of case report forms (CRF).The baseline characteristics, clinical/laboratory parameters were collected within 24 h of admission into the SICU from the electronic medical record system, including age, sex, Body Mass Index(BMI), patients sources, comorbidities, infection site, laboratory test results, methods of respiratory support, use of vasopressors or Continuous Renal Replacement Therapy (CRRT).Clinical scores included Lung injury prediction score (LIPS), Physiology and Chronic Health Evaluation II (APACHE II) and Sequential Organ Failure Assessment (SOFA).

### Statistical analysis

The data acquisition was carried out with Analyst 1.7.1 (ABsciex, MA, USA). Version 1.6 OS-Q (ABsciex, MA, USA) was employed for peak integration and quantification. IBM SPSS 27 (Armonk, New York, USA), R 4.3.0, and EZinfo (Waters, MA, USA) were employed for statistical analysis. Continuous variables were presented as mean ± standard deviation (SD) or median with quartiles 1 and 3 (Q1–Q3), and categorical variables were presented as percentiles. Continuous data between groups were compared using the t-test or Mann–Whitney U test, and categorical variables were compared using the chi-square test. For all analyses, statistical significance was indicated by a two-sided *p* < 0.05. We used multivariable generalized estimating equations with an independent correlation structure to estimate the association between urinary GAGs and the onset of ARDS, after accounting for the severity of the patients’ diseases and the levels of inflammatory factors in plasma. Correlated data were analyzed using Spearman correlation. The open-source tool MetaboAnalyst 5.0 (HYPERLINK: http://www.metaboanalyst.ca/) was also employed for statistical analysis and graph drawing.

## Results

**Fig. 3:**
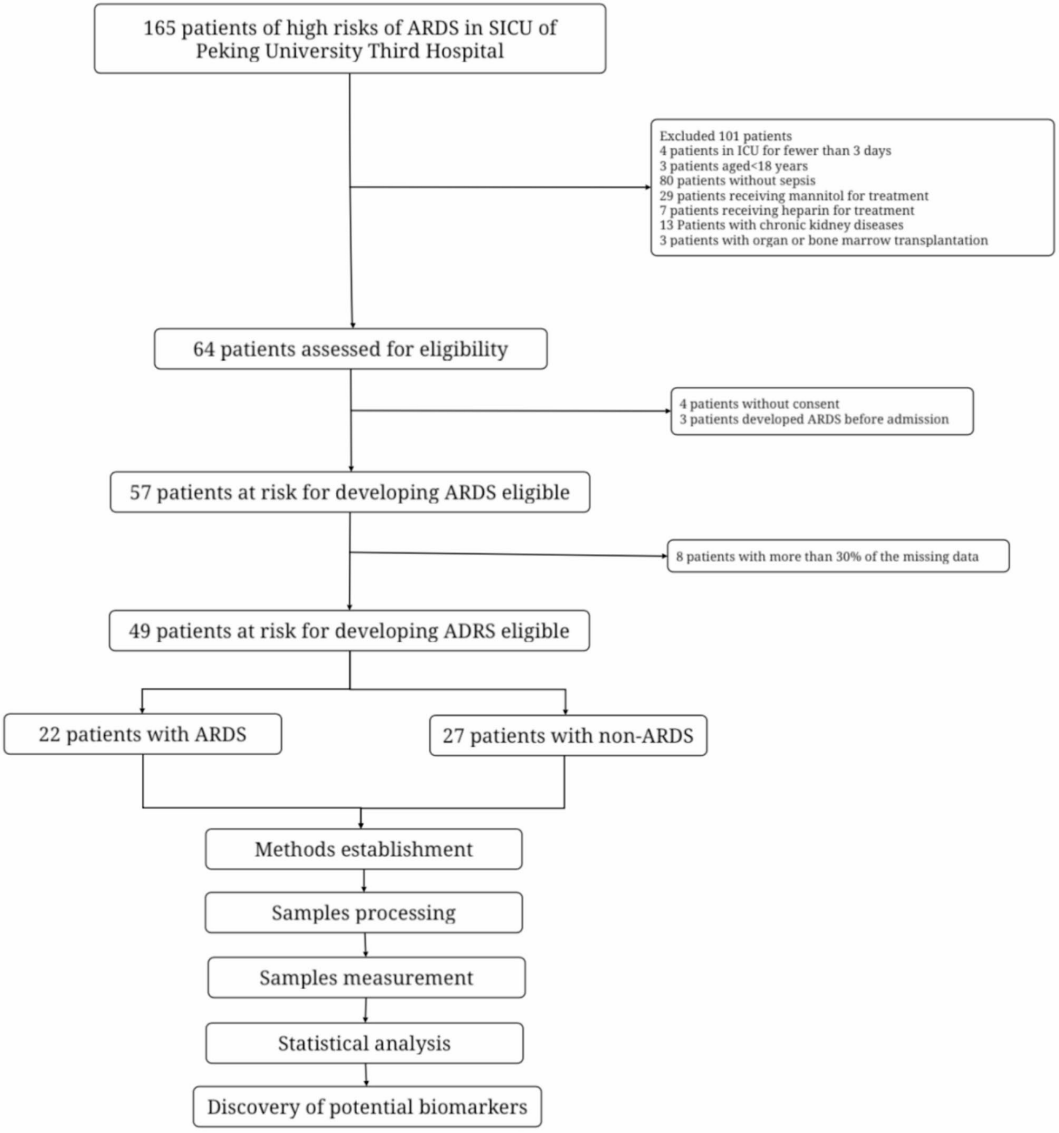
Flow chart of the study selection.22 patients with ARDS and 27 patients with non-ARDS were obtained.

During the study period, 165 patients who were admitted to the SICU of Peking University Third Hospital were screened. Among them, 4 patients stayed in the SICU for less than 3 days, 3 patients were aged < 18 years old, and 80 patients did not have sepsis. After excluding other ineligible patients, 49 patients were finally enrolled and were divided into 22 patients with ARDS and 27 patients with non-ARDS based on daily tracking of the patients’ conditions. Figure 3 shows the process of cohort selection.

### Baseline characteristics and clinical/laboratory parameters

In our cohort, there were 22 sepsis patients who developed ARDS. The comparison results showed that on the first day of admission, there were no significant differences between ARDS and non-ARDS patients in age, BMI, patient sources, hypertension, coronary heart disease, diabetes, malignant tumor, urinary tract infection, peritonitis, SOFA score, methods of respiratory support, use of vasopressors/CRRT, serum creatinine, or estimated glomerular filtration rate (eGFR). However, compared with non-ARDS patients, sex, pneumonia, APACHEII score, LIPS score, and PaO_2_/FiO_2_ showed statistical differences. As expected, sepsis patients with ARDS had a higher 28-day mortality after admission to the SICU: mortality rates were 6 (27.27%) vs. 1 (3.70%), *p* = 0.023, respectively. The baseline characteristics and clinical/laboratory parameters are shown in Table 1.

**Table 1:** Baseline characteristics and clinical/laboratory parameters.

Variable	ARDS(*n* = 22)	Non-ARDS(*n* = 27)	*P* value
Age (year) (Q1-Q3)	71.50 (64.00, 76.50)	64.00 (42.00, 76.00)	0.124
Sex (male) (%)	17 (77.27%)	11 (40.74)	0.019*
BMI (Q1-Q3)	24.05 (22.04, 25.64)	23.20 (21.30, 26 0.10)	0.666
Patients source			
Other department (%)	7 (31.82%)	5 (18.52%)	0.282
Emergency department and postoperation (%)	14 (63.64%)	19 (70.37%)	0.617
Elective postoperation (%)	1 (4.55%)	3 (11.11%)	0.404
Comorbidity			
Hypertension (%)	12 (54.55%)	10 (37.04%)	0.220
Coronary heart disease (%)	7 (31.82%)	3 (11.11%)	0.074
Diabetes (%)	4 (18.18%)	6 (22.22%)	0.727
Malignant tumor (%)	6 (27.27%)	3 (11.11%)	0.146
Infection site			
Pneumonia (%)	5 (22.73%)	1 (3.70%)	0.019*
Urinary tract infection (%)	0 (0.00%)	3 (11.11%)	0.107
Peritonitis (%)	17 (77.27%)	23 (85.19%)	0.477
APACHEII score (Mean ± SD)	21.09 ± 4.71	18.33 ± 4.67	0.046*
LIPS score (Mean ± SD)	9.34 ± 1.62	6.07 ± 2.61	0.000**
SOFA score (Mean ± SD)	8.77 ± 2.81	7.96 ± 2.16	0.260
Pa0_2_/ FiO_2_(Q1-Q3)	154.00 (114.50, 194.00)	310.00 (224.00, 388.00)	0.015*
Serum creatinine (Q1-Q3)	85.00 (70.00, 118.00)	85.00 (58.00, 153.00)	0.599
eGFR (Mean ± SD)	67.57 ± 28.88	73.93 ± 45.34	0.552
Methods of respiratory support			
Oxygen inhalation through the nasal tube(%)	5 (22.73%)	4 (14.81%)	0.477
Noninvasive mechanical ventilation (%)	2 (9.09%)	2(7.41%)	0.830
Invasive mechanical ventilation (%)	15 (68.18%)	22 (81.48%)	0.282
Noninvasive and invasive mechanical ventilation	4 (18.18%)	1 (3.70%)	0.096
Use of vasopressors (%)	20 (90.91%)	25 (92.59%)	0.830
Use of CRRT (%)	6 (27.27%)	8 (29.63%)	0.856
Outcome			
28-day mortality rate (%)	6 (27.27%)	1 (3.70%)	0.019*

* *p* < 0.05 ** *p* < 0.01.

### Urinary GaGs and plasma inflammatory factors of the first day in sepsis patients

**Fig. 4:**
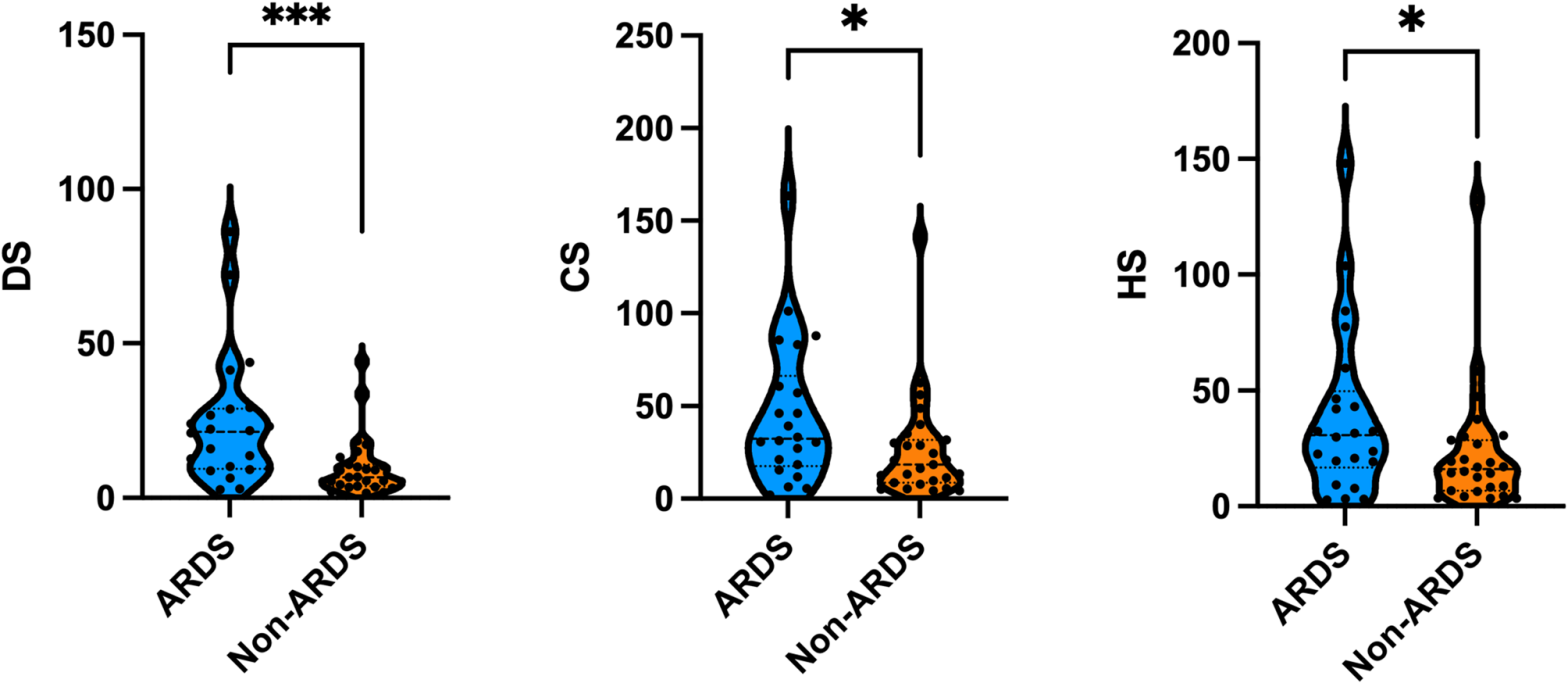
Urinary GAGs of the first day showed significant differences between two groups. * *p* < 0.05 ** *p* < 0.01.

Through continuous monitoring of the patients’ conditions and daily preservation of biological samples, we obtained a total of 49 patients’ time-series biological samples, including 132 plasma samples and 132 urine samples. On the first day of enrollment, among inflammatory factors, only TNF-α showed a significant difference between the two groups of patients. However, the levels of DS, CS, and HS in urine on the first day were higher in septic patients who later developed ARDS than in those who did not develop ARDS, and the differences were statistically significant. Details are shown in Table 2; Fig. 4.To address potential confounding by pulmonary infections, we conducted a sensitivity analysis by excluding patients with pneumonia (*n* = 6/49),and found that urinary DS, CS and HS of the first day still showed statistically significant differences when comparing ARDS versus non-ARDS patients (Mann-Whitney U test, DS: *p* = 0.001***; CS: *p* = 0.010**; HS: *p* = 0.017*).

**Table 2:** Urinary GaGs and plasma inflammatory factors of the first day between two groups.

Variable	ARDS	Non-ARDS	*P* value
DS	21.45 (9.49–28.96)	6.66 (3.82–10.80)	0.001**
CS	32.33 (17.66–66.39)	18.40 (8.61–31.70)	0.027*
HS	30.76 (16.76–49.76)	16.10 (6.75–28.58)	0.037*
IL-1β	8.82 (2.51–24.28)	17.36 (9.65–100.96)	0.093
IFN-γ	12.86 (2.97–26.02)	5.40 (1.87–16.91)	0.188
TNF-α	35.78 (11.84–77.32)	15.32 (9.24–35.83)	0.027*
MCP-1	283.56 (130.21–663.79)	460.04 (236.17–2103.95)	0.159
IL-6	331.01 (93.62–1475.63)	282.41 (125.93–1190.90)	0.717
IL-8	101.95 (46.57–180.56)	104.24 (36.11–915.81)	0.763
IL-10	36.20 (16.49–135.83)	46.19 (7.41–107.61)	0.904
AngII	2491.55 (1318.17–4635.21)	3164.35 (1495.33–5167.23)	0.220
KL-6	30.90 (27.02–52.14)	31.45 (27.07–37.50)	0.615
RAGE	273.81 (203.13–380.84)	288.28 (149.91–387.19)	0.482

* *p* < 0.05 ** *p* < 0.01.

### Relevance between urinary GaGs and clinical parameters

**Fig. 5:**
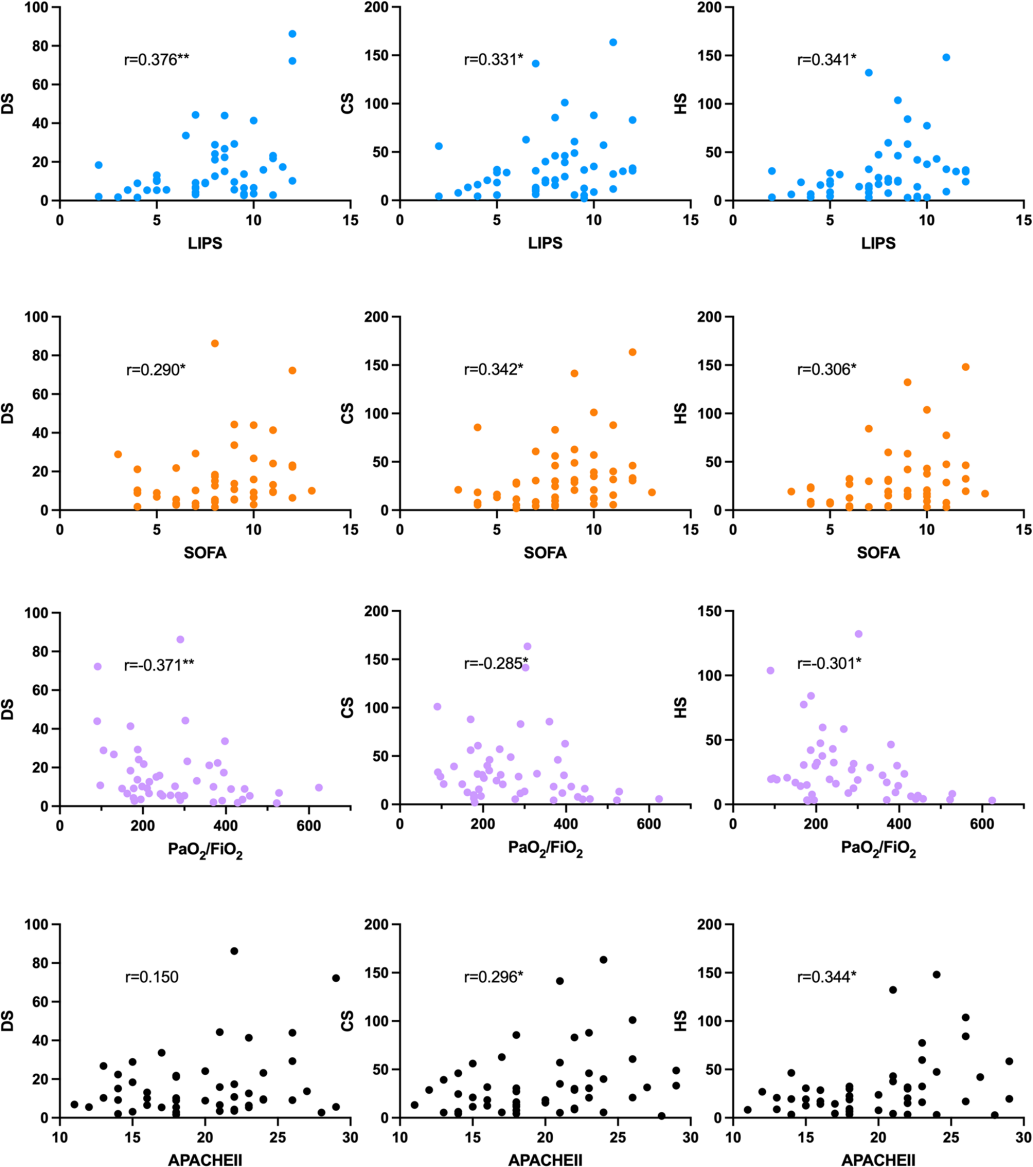
Relevance between urinary GAGs and clinical parameters. * *p* < 0.05 ** *p* < 0.01.

Spearman correlation analysis showed that on the first day, urinary DS, CS, and HS levels correlated with PaO_2_/FiO_2_, LIPS, and SOFA scores, as well as with APACHE II (except for DS) (details are shown in Fig. 5), indicating that urinary GAGs might reflect the severity of lung injury, organ dysfunction, and disease severity.

### Parameters screening related to the occurrence of ARDS in patients with sepsis using generalized estimating equation

**Fig. 6:**
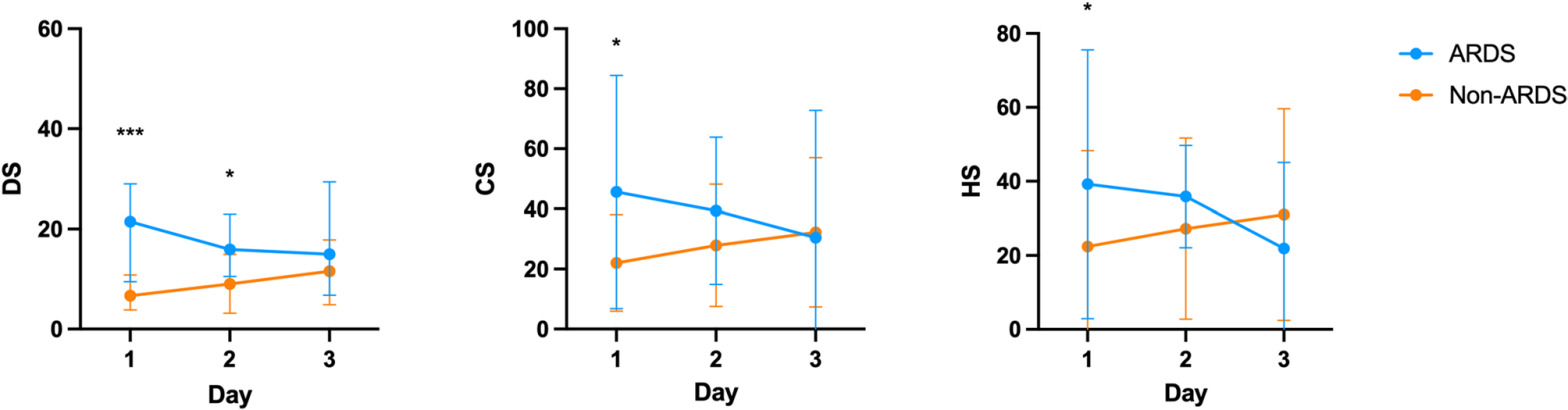
The variation trends of the three GAGs during the initial three days. * *p* < 0.05 ** *p* < 0.01***p < 0.001.

Because we conducted multiple measurements based on time series for each patient’s sample, all variables showing significant differences in Tables 1 and 2 were enrolled in multivariable generalized estimating equation. After matching for age, pneumonia, APACHE II, LIPS, and PaO_2_/FiO_2_, DS and TNF-α were shown to be independently associated with the future development of ARDS(Table 3). The variation trends of the three GAGs during the initial three days are additionally displayed in the Fig. 6.

**Table 3:** Results of multivariable generalized estimating equation.

Variable	B	Standard error	95% CI	Wald χ2	*P* value	OR	95% CI
Sex	0.793	0.8097	– 0.794 to 2.38	0.958	0.328	2.209	0.452 to 10.801
Pneumonia	2.507	1.3425	– 5.139 to 0.124	3.488	0.062	0.081	0.006 to 1.132
APACHEII	0.154	0.1025	– 0.047 to 0.355	2.252	0.133	1.166	0.954 to 1.426
LIPS	0.082	0.1785	– 0.268 to 0.431	0.209	0.648	1.085	0.765 to 1.539
PaO_2_/FiO_2_	0.009	0.0054	– 0.002 to 0.019	2.477	0.116	1.009	0.998 to 1.019
DS	0.087	0.0314	0.026 to 0.149	7.69	0.006**	1.091	1.026 to 1.16
CS	-0.002	0.0274	– 0.056 to 0.051	0.007	0.931	0.998	0.946 to 1.053
HS	0.007	0.0272	– 0.046 to 0.061	0.071	0.79	1.007	0.955 to 1.062
TNF-α	0.024	0.0089	0.007 to 0.042	7.334	0.007**	1.024	1.007 to 1.042

* *p* < 0.05 ** *p* < 0.01.

## Discussion

Our study found that in sepsis patients, increased urinary GAGs and plasma TNF-α levels were associated with subsequent ARDS development. Even after adjusting for patients’ baseline characteristics and initial lung injury severity, urinary DS and plasma TNF-α levels remained independently associated with ARDS occurrence. Therefore, urinary GAGs may represent novel biomarkers for ARDS prediction, and future studies could explore combining urinary GAGs with inflammatory factors to improve ARDS predictive accuracy.

Although the primary sources of infection in sepsis (e.g., pneumonia, peritonitis, pancreatitis) may differ, emerging evidence suggests that glycocalyx degradation follows a shared pathway in ARDS development^17^. In sepsis, the endothelial glycocalyx undergoes progressive degradation through multiple interconnected pathways that ultimately contribute to the development of acute respiratory distress syndrome (ARDS). The process begins when pathogen-associated molecular patterns (PAMPs) and damage-associated molecular patterns (DAMPs) activate Toll-like receptors on immune cells, triggering a cytokine storm characterized by elevated TNF-α, IL-1β, and IL-6^18^. These inflammatory mediators induce glycocalyx breakdown through enzymatic cleavage by upregulating Heparanase(HPA) and matrix metalloproteinases (MMPs)^19,20^, particularly MMP-9, which specifically target HS side chains and core proteins like syndecan-1^21^. Concurrently, nicotinamide adenine dinucleotide phosphate (NADPH) oxidase-derived reactive oxygen species (ROS) cause oxidative fragmentation of GAGs polysaccharide chains, while hemodynamic disturbances from microcirculatory failure led to mechanical stripping of the glycocalyx. The shed GAG components, then propagate lung injury through several mechanisms: loss of the glycocalyx barrier exposes endothelial junctions (e.g., VE-cadherin) and increases vascular permeability; Circulating HS potentiates vascular endothelial growth factor (VEGF)-mediated leakage; Unmasked adhesion molecules promote neutrophil recruitment; Shed DS acts as a DAMP that activates Toll-Like Receptor 4(TLR4) on neutrophils, triggering neutrophil extracellular trap (NET) release and further inflammation. Additionally, depletion of HS-bound antithrombin III (ATIII) sites disrupts thrombin regulation, leading to microthrombosis that compounds pulmonary vascular injury^4^. The pulmonary vasculature is particularly vulnerable to these effects due to its unique anatomical and physiological characteristics - the immense alveolar-capillary surface area (~ 70 m^2^), exceptionally thin endothelial layer (0.1–0.2 μm), and low-pressure circulation make the lung highly dependent on an intact glycocalyx for maintaining barrier function^17^. This vulnerability explains why systemic glycocalyx degradation, as reflected by urinary GAG excretion, often manifests first and most severely as ARDS, even when the primary infection originates outside the lungs.

Previously, Murphy’s research showed that the extent of endothelial glycocalyx degradation (measured by a component named Syndecan 1) is associated with non-pulmonary organ dysfunction in subjects with sepsis and is associated with ARDS but only in the subgroup with non-pulmonary sepsis^22^. In our study, we used LC-MS to measure urinary GAGs in time-series samples and found that the onset of ARDS in all sepsis patients was associated with an early increase in urinary GAGs, especially DS, compared with non-ARDS sepsis patients. Research has confirmed that different pathophysiologic insults produce characteristic patterns of released glycocalyx fragments^23^, and different types of GAGs may have different shedding periods. Although HS tends to be emphasized as the most biologically active glycosaminoglycan, and it has been discovered that pulmonary endothelial glycocalyx degradation increases neutrophil adhesion and propagates lung injury in a Heparanase-2 dependent fashion^24^, heparin is largely confined to mast cells, whereas DS is a ubiquitous element of the extracellular matrix^25^. Growing evidence suggests DS is an important co-factor in a variety of cell behaviors like cardiovascular disease, tumorigenesis, infection, wound repair, and fibrosis. What’s more, Schmidt used HS and CS to predict renal dysfunction in septic shock and ARDS patients, showing that patients who later developed AKI had significantly elevated urinary total glycosaminoglycans at Day 0 and 3, largely derived from consistently elevated urinary CS^13^. CS and DS are stereoisomers and therefore have similar structures^26^. In our research, DS performed better in predicting ARDS, which might suggest that CS is mainly related to kidney injury^27,28^.

In addition, we found correlations between urinary GAGs and clinical parameters (PaO_2_/FiO_2_, LIPS, SOFA, and APACHE II) on the first day after admission to SICU, showing that urinary GAGs could reflect pulmonary and other organ dysfunction, indicating urinary GAGs have a close correlation with organ function.

Then, it was found that urinary GAGs and related sulfated disaccharide indexes in ARDS patients were related to hospital mortality^13^. In our study, 28-day mortality differed between ARDS and non-ARDS patients, but we did not focus on the predictive value of urinary GAGs for mortality. Further studies should be conducted to determine whether they could be predictive biomarkers of mortality in sepsis patients.

When it comes to inflammatory factors, inflammatory storm is considered as one of the important causes leading to the occurrence of ARDS. In recent years, more and more studies have attempted to predict the occurrence of ARDS using inflammatory factors in plasma. Meanwhile, studies have also shown that there is an interaction between inflammatory factors and GAGs. Fahlgren found that IFN-γ-induced cell maturation would lead to more carcinoembryonic antigen (CEA) and carcinoembryonic antigen-related cell adhesion molecule 6 (CEACAM6, localized to the apical glycocalyx of normal colonic epithelium and suggested to play a role in innate immunity) in glycocalyx layers. This would be helpful from the point of view of immune defense, because bacteria would be entrapped at a distance from the epithelial cell surface, providing further protection^29^. The mast cell IFN-γ binding element was identified as DS^30^. GAGs are known to bind IFN-γ to protect it from degradation and increase its half-life, prolonging its pro-inflammatory effects^31^. TNF-α is also a central factor in cytokine storm. Schmidt’s research showed that TNF-α was one of the factors that could damage the glycocalyx during inflammatory diseases through HPA^32^, and studies have shown that shedding of the glycocalyx decreased with etanercept (TNF-α antagonist) in adult volunteers^33^. Chemokines and GAGs interact to localize chemokines to inflammatory environments where they are produced^34^. IL-8 also binds CS/DS in arthritis, acute nephritis, sepsis, and ischemia-reperfusion injury in brain, etc^35^. Ang II is also a purported causative agent for endothelial glycocalyx breakdown^36^. Lukasz found that Ang II treatment causes rapid degradation of endothelial glycocalyx both in vivo and in vitro, using a human umbilical vein endothelial cell line and mice^36^. Urinary GAG levels correlated significantly with plasma Ang II in Falciparum malaria^37^. RAGE was found to be associated with airspace GAG levels^9^, and DS, CS and HS might play roles in RAGE-mediated cell signaling, tumor metastasis, and other biological phenomena^38^. More research is needed to find out the interaction mechanism between inflammatory factors and GAGs.

We know that vascular glycocalyx is one of the earliest sites influenced by inflammation^39^. The function of the glycocalyx depends on its structural integrity. In sepsis, the glycocalyx sheds due to inflammation, leading to a series of pathological changes, including increased vascular permeability, changes in vascular tension, disorder of the coagulation system and antioxidant system, and adhesion between leukocytes and vascular endothelial cells^39^.

Notably, GAGs, AngII, and RAGE are all biomarkers reflecting endothelial or epithelial cell injury, but compared to Ang II and RAGE, GAGs offer distinct advantages for early ARDS recognition, particularly due to their direct association with endothelial glycocalyx degradation—a process that precedes clinical manifestations of lung injury^40^. While Ang II primarily reflects generalized endothelial activation and RAGE indicates established alveolar epithelial damage, urinary GAGs (especially DS and HS) serve as real-time indicators of vascular barrier disruption, detectable even before overt hypoxemia develops^41^.Key strengths of GAGs include temporal sensitivity: GAG shedding occurs early in the “two-hit” model of ARDS (initial endothelial injury followed by inflammation), whereas Ang II peaks later during cytokine storms, and RAGE rises predominantly after epithelial injury; Organ Specificity: Unlike Ang II (systemic endothelial dysfunction), GAGs like DS show stronger correlations with pulmonary vascular injury (e.g., higher LIPS scores and PaO_2_/FiO_2_ decline); Non-Invasive Monitoring: Urinary GAGs enable serial assessments without invasive procedures (e.g., Bronchoalveolar lavage or plasma for RAGE)^40^, critical for ICU patients. Thus, while Ang II and RAGE remain valuable for disease staging, GAGs provide a unique window into the earliest phases of ARDS pathogenesis, potentially enabling preemptive interventions.

Based on the above background, in this study, we attempted to combine GAGs with inflammatory factors to observe their relationship with ARDS occurrence. In the univariate analysis, urinary DS, CS, HS, and plasma TNF-α were associated with ARDS occurrence. When these factors were simultaneously included in the generalized estimating equation along with baseline data that showed significant differences, we found that only urinary DS and plasma TNF-α were independently associated with ARDS onset in sepsis patients.

It should be emphasized that our study population differs from traditional ARDS cohorts in medical ICUs, as we specifically focused on surgical patients with sepsis. This population represents a unique clinical entity where the pathophysiology of ARDS may be influenced by both the primary infectious process and surgical trauma. This study observed a relatively high proportion of peritonitis as the primary infection source, which differs from traditional ARDS studies where pulmonary infections predominantly prevail. However, this distribution pattern closely aligns with epidemiological data on sepsis in surgical ICUs, multiple studies report that intra-abdominal infections account for 40–60% of sepsis cases in SICU populations^42^.This discrepancy precisely reflects the unique characteristics of surgical patients with sepsis.

Taking all the findings into consideration, GAGs may be a novel therapeutic target in sepsis and ARDS. The treatment directions can be divided into inhibiting the degradation of GAGs and promoting their regeneration. Some studies have focused on therapy targeting the endothelial glycocalyx, such as therapeutic plasma exchange, glucocorticoids, heparinoid preparations, and vascular endothelial growth factor, etc^43–46^. Further research is needed to establish a complete, highly specific preventive and treatment strategy for ARDS.

Our study is the first to evaluate the predictive value of DS, CS and HS for ARDS in sepsis patients. Our research was performed using an LC-MS system. Another study has shown that LC-MS results could be largely replicated using an inexpensive, rapidly performed colorimetric assay of sulfated glycosaminoglycans^13^.Preliminary data suggest urinary GAGs as candidate biomarkers, but their clinical utility requires validation in independent cohorts.

Although strict inclusion and exclusion criteria were used in the present study to find correlation between onset of ARDS and urinary GAGs in sepsis patients, our study had several limitations. (1) The sample size was small, and we did not analyze HA or KS, which may have additional predictive value. However, our pilot data on DS/CS/HS provide a foundation for subsequent expanded panels. While our study identified urinary GAGs as potential ARDS biomarkers, we acknowledge that the mixed etiology and different severity levels of our cohort may introduce confounding effects. We need more samples and multicenter studies to expand and verify our conclusions. (2) We only identified novel biomarkers associated with ARDS occurrence but did not establish a predictive model for ARDS. (3) More basic experiments are needed to investigate the correlation between urinary GAGs and plasma inflammatory factors. Nevertheless, our study has several strengths: (1) No previous studies have focused on the predictive value of urinary DS for ARDS; (2) Samples collected before ARDS diagnosis made our predictive results more reliable; (3) We established time-series data by collecting samples consecutively and used generalized estimating equations instead of logistic regression to account for temporal correlations among repeated laboratory measurements.

## Conclusions

In our dynamic study cohort, we found an association between levels of urinary DS, CS, and HS measured by LC-MS and subsequent ARDS onset in sepsis patients. Urinary GAGs correlated with PaO_2_/FiO_2_, LIPS, SOFA, and APACHEII scores. DS and TNF-α show promise as early ARDS biomarkers in septic patients, but their generalizability across ARDS subtypes requires further validation. Future multicenter studies should prioritize etiology-specific enrollment to establish clinical utility.

## Electronic supplementary material

Below is the link to the electronic supplementary material.

Supplementary Material 1

## Data Availability

The datasets generated during and/or analysed during the current study are available from the corresponding author on reasonable request.

## Abbreviations

ARDS: Acute respiratory distress syndrome
GAGs: Glycosaminoglycans
LIPS: Lung injury prediction score
ALI: Acute lung injury
CS: Chondroitin sulfate
DS: Dermatan sulfate
HS: Heparan sulfate
KS: Keratan sulfate
HA: Hyaluronan
SICU: Surgical intensive care unit
IFN-γ: Interferon-γ
TNF-α: Tumor necrosis factor-α
MCP-1: Monocyte chemoattractant protein-1
IL-6: Interleukin-6
IL-8: Interleukin-8
IL-10: Interleukin-10
AngII: AngiopoietinII
KL-6: Krebs von den Lungen-6
RAGE: Receptor for advanced glycation end products
CRF: Case report forms
APACHE II: Acute physiology and chronic health evaluation II
SOFA: Sequential organ failure assessment
SD: Standard deviation
CI: Confidence interval
eGFR: Estimated glomerular filtration rate
PAMPs: Pathogen-associated molecular patterns
DAMPs: Damage-associated molecular patterns
HPA: Heparanase
MMPs: Matrix metalloproteinases
NADPH: Nicotinamide adenine dinucleotide phosphate
ROS: Reactive oxygen species
VEGF: Vascular endothelial growth factor
TLR4: Toll-Like Receptor 4
NET: Neutrophil extracellular trap
AT III: Antithrombin III
CEA: Carcinoembryonic antigen
