# Supplementary material for: Associations between urinary glycosaminoglycans and onset of acute respiratory distress syndrome in sepsis patients: a prospective exploratory study
Source: Sci Rep. 2025 May 22;15:17783. doi: 10.1038/s41598-025-02109-5 (PMC12098999; doi:10.1038/s41598-025-02109-5)
Supplement: Supplementary file 1 — Supplementary Material 1 [file 41598_2025_2109_MOESM1_ESM.doc]

**Supplementary Table1 Gradient Elution program for GAGs analysis.A:10mM acetic acid aqueous solution;B:acetonitrile**

| Time/min | A% | B% |  |
| --- | --- | --- | --- |
| 0 | 90 | 10 |  |
| 0.5 | 90 | 10 |  |
| 4 | 70 | 30 |  |
| 4.01 | 70 | 30 |  |
| 5 | 70 | 30 |  |
| 5.01 | 90 | 10 |  |
